# Establishing a network of specialist Porphyria centres - effects on diagnostic activities and services

**DOI:** 10.1186/1750-1172-7-93

**Published:** 2012-12-10

**Authors:** Mette C Tollånes, Aasne K Aarsand, Jørild Haugen Villanger, Egil Støle, Jean-Charles Deybach, Joanne Marsden, Jordi To-Figueras, Sverre Sandberg

**Affiliations:** 1 Norwegian Porphyria Centre (NAPOS), Laboratory of Clinical Biochemistry, Haukeland University Hospital, Bergen, Norway AND Institute of Public Health and Primary Health Care, University of Bergen, Bergen, Norway; 2 Norwegian Porphyria Centre (NAPOS), Laboratory of Clinical Biochemistry, Haukeland University Hospital, Bergen, Norway; 3 Norwegian Porphyria Centre (NAPOS), Laboratory of Clinical Biochemistry, Haukeland University Hospital, Bergen, Norway; 4 Norwegian Porphyria Centre (NAPOS), Laboratory of Clinical Biochemistry, Haukeland University Hospital, Bergen, Norway; 5 Assistance Publique-Hôpitaux de Paris, Centre Français des Porphyries, Hôpital Louis Mourier, Colombes, France AND Centre de Recherche Biomedicale Bichat-Beaujon, Université Paris Diderot, Paris, France; 6 Department of Clinical Biochemistry, King's College Hospital NHS Foundation Trust, Denmark Hill, London, UK; 7 Biochemistry and Molecular Genetics Unit, Hospital Clínic, IDIBAPS, University of Barcelona, Barcelona, Spain; 8 Norwegian Porphyria Centre (NAPOS), Laboratory of Clinical Biochemistry, Haukeland University Hospital, Bergen, Norway AND Norwegian Quality Improvement of Primary Care Laboratories (NOKLUS), Section for General Practice, University of Bergen, Bergen, Norway

**Keywords:** Activity data, Diagnostics, Metabolic disease, Porphyria, Specialist centre

## Abstract

**Background:**

The porphyrias are a heterogeneous group of rare metabolic diseases. The full spectrum of porphyria diagnostics is usually performed by specialized porphyria laboratories or centres. The European Porphyria Initiative (EPI), a collaborative network of porphyria centres formed in 2001, evolved in 2007 into the European Porphyria Network (EPNET), where participating centres are required to adhere to agreed quality criteria. The aim of this study was to examine the state and distribution of porphyria diagnostic services in 2009 and to explore potential effects of increased international collaboration in the field of these rare diseases in the period 2006–2009.

**Methods:**

Data on laboratory, diagnostic and clinical activities and services reported to EPI/EPNET in yearly activity reports during 2006 through 2009 were compared between reporting centres, and possible time trends explored.

**Results:**

Thirty-five porphyria centres from 22 countries, five of which were non-European associate EPNET members, filed one or more activity reports to EPI/EPNET during the study period. Large variations between centres were observed in the analytical repertoire offered, numbers of analyses performed and type and number of staff engaged. The proportion of centres fulfilling the minimum criteria set by EPNET to be classified as a specialist porphyria centre increased from 80% to 94% during the study period.

**Conclusions:**

Porphyria services are unevenly distributed, and some areas are probably still lacking in specialized porphyria services altogether. However, improvements in the quality of diagnostic services provided by porphyria centres participating in EPI/EPNET were observed during 2006 through 2009.

## Background

The porphyrias are a heterogeneous group of rare metabolic diseases caused by abnormal function in one of the eight enzymes of the haem biosynthetic pathway, leading to overproduction and accumulation of haem precursors [1]. Symptoms of disease can present as acute attacks of abdominal pain and neuropsychiatric symptoms (acute intermittent porphyria (AIP) and δ-aminolevulinic acid (ALA) dehydratase deficiency), cutaneous symptoms (porphyria cutanea tarda (PCT), erythropoietic protoporphyria (EPP), X-linked dominant protoporphyria (XLDPP) and congenital erythropoietic porphyria (CEP)) or both (hereditary coproporphyria (HCP) and variegate porphyria (VP)). Acute attacks can be triggered by various factors such as many commonly used drugs, alcohol, hormonal changes, infectious disease and fasting, while cutaneous symptoms are triggered by exposure to light. In symptomatic patients, diagnosis depends on the biochemical detection of haem precursor accumulation in blood, urine and faeces [1]. The three most common acute porphyrias, AIP, VP and HCP, are inherited in an autosomal dominant fashion with low clinical penetrance, and presymptomatic DNA testing is often offered for identification of healthy at-risk relatives. Because of the complexity of diagnosing these disorders, the full spectrum of porphyria diagnostics is in most countries performed by specialized porphyria laboratories or centres, but general laboratories might offer some front-line porphyrin analyses. The porphyrias are probably under-diagnosed, as are many other rare inherited metabolic diseases [2], and the availability and quality of porphyria diagnostics and services might vary both within and between countries.

The European Porphyria Initiative (EPI) was formed in 2001 as a collaborative network of porphyria centres in Europe, with non-European centres participating as associate EPI members [3]. In 2007, the work of EPI was further continued and expanded and the European Porphyria Network (EPNET) was formed [4]. EPNET was established, initially as a three-year project funded by the European Commission through its Public Health and Consumer Protection Directorate, with the aim of setting up an effective European network consisting of specialist porphyria laboratories and diagnostic centres adhering to agreed quality criteria [5]. EPNET began its work by focusing on seven work packages (WP), of which the first three had administrative purposes. The aims of WP6 were to collect data about the European specialist porphyria centres’ activities and workload and give feedback to the centres comparing their activity levels to the others’, to set up a clinical and analytical external quality assessment (EQA) scheme, to develop uniform quality standards and to draw up consensus-agreed protocols for porphyria diagnostics and monitoring.

The aim of the present study was to use the yearly reported activity data collected as a part of the EPNET project’s WP6 to examine the state and distribution of porphyria diagnostic services among participating centres, and to explore potential effects of increased international collaboration in the field of rare diseases.

## Methods

Since 2003/2004, EPI/EPNET centres and associate non-European members have been required to submit yearly reports with data on the number of biochemical and genetic laboratory analyses performed, individuals investigated and/or request forms received, new diagnoses made, type of clinical services provided, description of staff involved, participation in external quality assessment (EQA) programmes and accreditation/certification status of the laboratory facilities. New diagnoses reported include the number of new symptomatic porphyria patients as well as the number of presymptomatic cases identified, i.e. mutation carriers identified based on DNA testing of healthy at-risk relatives. From 2005 onwards, the centres have been evaluated with regard to whether their services fulfil an agreed set of quality criteria. These include a) the ability to perform the following biochemical analyses: in urine; ALA, porphobilinogen (PBG) and total porphyrins with fractionation, in faeces; total porphyrins with fractionation including separation of coproporphyrin isomers I and III, in blood; erythrocyte protoporphyrin and plasma fluorescence scanning, b) participation in a relevant EQA programme (from 2009 onwards), and c) providing laboratory reports that include a detailed interpretation of the laboratory results and incorporate expert clinical advice. Since 2006, individual feedback reports have been provided to the centres, comparing each centre’s level of activity to that of the other participating centres.

In the present study, the summary statistics of the 2009 feedback reports were used to examine the state and distribution of porphyria diagnostic activities and services this year. For the 12 countries where the participating porphyria centres reported that they provide national coverage of porphyria diagnostics in 2009, and where all the relevant data had been reported, numbers of individuals investigated per 100,000 inhabitants and the numbers of new AIP and PCT diagnoses per 100,000 inhabitants were also calculated. In addition, data from the 2006–2009 feedback reports were used to investigate trends and developments in laboratory analyses and diagnostic services offered, number of centres fulfilling the EPNET specialist porphyria centre criteria as well as numbers of new porphyria diagnoses reported in Europe.

## Results

A total of 35 porphyria centres from 22 countries, five of which were non-European, reported their activity data to EPNET during the study period (Table 1). However, not all centres reported data each year, nor did all centres respond to every question when participating.

**Table 1:** Porphyria centres providing activity data to EPNET from 2006 through 2009

**Country**	**Porphyria centre**	**2006**	**2007**	**2008**	**2009**
Australia	NSW Porphyria Reference Unit, Royal Prince Alfred Hospital, Camperdown	X	X	X	X
Belgium	Centre Belge des Porphyries, Hôpital Erasme - ULB, Brussels	X	X	X	X
	Metabool Centrum, UZ Gasthuisberg, Leuven	X	X	X	X
Brazil	Clinics Hospital of Ribeirao Preto, São Paulo			X	
Czech Republic	National Laboratory for Porphyric Disease, 1st Medical Faculty, Charles University of Prague	X	X	X	X
Denmark	Danish Porphyria Center, Viborg Regional Hospital	X	X	X	X
Finland	Porphyria Research Centre in Finland, University Central Hospital of Helsinki	X	X		X
France	Centre Francais des Porphyries, Hospital Louis Mourier (APHP), Colombes	X	X	X	X
Germany	Deutsches Kompetenz-Zentrum für Porphyriediagnostik und Konsultation, MVZ Labor Prof. Seelig, Karlsruhe	X	X	X	X
	Hospital of the University of Munich	X	X	X	X
	Porphyria Specialist Center Düsseldorf, University Hospital Düsseldorf	X	X	X	X
	Porphyria Center Saxony, Klinikum Chemnitz gGmbH	X	X	X	X
Hungary	Hungarian Porphyria Center, Ministry of Defence National Health Center, Budapest		X	X	X
	Porphyrin Laboratory, University of Szeged	X	X	X	X
Ireland	Irish Porphyria Specialist Centre, St. James´s Hospital, Dublin	X	X	X	X
Israel	National Service for the Biochemical Diagnosis of Porphyrias, Rabin Medical Centre, Beilinson Hospital, Petah Tikva	X	X	X	X
Italy	U.O. Medicina Interna Ia, IRCCS Ca' Granda Ospedale Maggiore Policlinico, University of Milan	X	X	X	X
	Laboratorio di Diagnostica Delle Porfirie e Delle Aminoacidopatie, Dipartimento integrato di Medicine e Specialita Mediche, University of Modena and Reggio Emilia	X	X	X	X
	Porphyria Centre and Hereditary Metabolic Diseases, San Gallicano Institute IRCCS Rome	X	X	X	X
	Interregional Reference Centre for prevention, surveillance, diagnosis and therapy of porphyrias, "Casa Sollievo della Sofferenza" Hospital IRCCS, San Giovanni Rotondo (Foggia)				X
The Netherlands	Euregional Porphyria Center Maastricht (EPCM), Maastricht University Medical Center	X			
	Porphyria Center Rotterdam, Erasmus Medical Center, Rotterdam	X	X	X	X
New Zealand	Porphyria centre, Canterbury Health Laboratories, Christchurch		X	X	X
Norway	Norwegian Porphyria Centre (NAPOS), Haukeland University Hospital, Bergen	X	X	X	X
Poland	Laboratory of Porphyria, Institute of Hematology and Transfusion Medicine (IHiT), Warsaw	X	X	X	X
South Africa	UCT Lennox Eales Porphyria Labs, UCT Medical School, Cape Town	X	X	X	X
Spain	Porphyria Unit, Hospital Clinic, University of Barcelona	X	X	X	X
	Porphyria Unit, Hospital Universitario Doce de Octubre, Madrid	X	X	X	X
Sweden	Porphyria Centre Sweden, Karolinska University Hospital, Stockholm	X	X	X	X
Switzerland	Porphyrie-Referenzlabor, Stadtspital Triemli Zürich	X	X	X	X
United Kingdom	Cardiff SAS Porphyria Service, University Hospital of Wales, Cardiff	X	X	X	X
	Leeds Teaching Hospitals NHS Trust, Leeds		X	X	X
	Porphyria Laboratory, King’s College Hospital, London	X	X	X	X
	Salford Royal NHS Foundation Trust, Salford	X	X	X	X
USA	Porphyria Center, University of Texas Medical Branch, Galveston	X	X	X	X

With regard to laboratory services offered, urinary ALA and PBG were the only two biochemical analyses offered and performed by all responding centres every year (Table 2). Urinary PBG is the primary front-line analysis for diagnosing the three most common acute porphyrias (AIP, VP and HCP), but plasma fluorescence scanning and estimation of the faecal coproporphyrin III: I ratio must be performed to adequately differentiate between them [6]. During the study period, the proportion of reporting centres that were able to, and had performed faecal porphyrin fractionation including coproporphyrin isomer I and III differentiation, increased from 77% to 85% (Table 2). Similarly, there was an increase in the proportion of responding centres performing erythrocyte protoporphyrin analyses with the distinction between metal free and zinc-chelated protoporphyrin, from 67% in 2006 to 79% in 2009. Eighty-three percent of reporting centres had performed plasma fluorescence scanning in 2006 compared to 88% in 2009. The proportion of centres preforming DNA- analyses, however, was relatively stable over time.

**Table 2:** Number of participating EPNET centres and associate members having performed diagnostic laboratory analyses during 2006 through 2009

**Material**	**Analyte**	**2006**	**2007**	**2008**	**2009**
		**(30 centres)**	**(32 centres)**	**(32 centres)**	**(33 centres)**
Urine	ALA^a^	30	32	32	33
	PBG^a,b^	30	32	32	33
	Total porphyrins^a^	25	27	28	29
	Fractionation of porphyrins^a,c^	30	30	31	31
Faeces	Total porphyrins^a^	22	21	23	26
	Fractionation of porphyrins with separation of copro I/III^a,b,c^	23	25	28	28
	Fractionation of porphyrins without separation of copro I/III	9	5	5	5
Plasma	Plasma fluorescence scanning^a,b,c^	25	29	29	29
	Total porphyrins	11	13	15	16
	Fractionation of porphyrins	16	9	11	11
	ALA	not asked	not asked	not asked	3
	PBG	not asked	not asked	not asked	3
Whole blood	Erythrocyte total protoporphyrin^a,d^	23	27	28	30
	Erythrocyte free and zinc-chelated protoporphyrin ^a,d^	20	24	23	26
	Porphobilinogen deaminase activity	20	22	22	23
	Uroporphyrinogen decarboxylase activity	12	9	7	10
	Other enzyme analyses^e^	12	12	8	10
DNA analyses	Hydroxymethylbilane synthase gene	19	16	18	17
	Uroporphyrinogen III synthase gene	8	3	1	9
	Uroporphyrinogen III decarboxylase gene	13	10	10	12
	Coproporphyrinogen III oxidase gene	11	13	13	12
	Protoporphyrinogen oxidase gene	19	17	18	17
	Ferrochelatase gene	16	13	14	15
	ALA synthase 1 gene	not asked	not asked	0	1
	ALA synthase 2 gene	not asked	not asked	4	7

^a^ Minimum laboratory analyses required for being classified as an EPNET specialist porphyria centre.

^b^ Considered necessary for the diagnosis and discrimination of the three most common acute porphyrias (acute intermittent porphyria, hereditary coproporphyria and variegate porphyria).

^c^ Considered necessary for the diagnosis and discrimination of cutaneous porphyrias with active skin lesions (not including EPP).

^d^ Two out of three (total, free and/or zink-chelated protoporphyrin) considered necessary for the diagnosis of EPP.

^e^ Other enzymes comprise ALA dehydratase, ALA synthase, uroporphyrinogen III synthase, coproporphyrinogen oxidase, protoporphyrinogen oxidase and ferrochelatase.

Urinary PBG was the most frequently performed laboratory analysis in 2009, with centres performing between 23 and 1342 (median 255) analyses (data not shown). Nine out of 33 reporting centres analysed less than 200 PBG samples, whereas five laboratories analysed more than 1000 samples. Urinary total porphyrins was the second most frequently performed analysis, with half of the centres performing this analysis more frequently than PBG. Twenty-eight out of the 33 reporting centres were able to perform one or more enzyme activity analysis, and 19 offered DNA analysis for one or more porphyria diseases.

With regard to human resources available to the participating porphyria centres in 2009, the median number of whole time equivalent (WTE) employees was 3.9 (range 0.3-10.7). The majority of the employees consisted of medical laboratory technologists, biomedical scientists and technicians. Four centres had less than one WTE employee, while seven centres had eight or more. Thirty out of 33 centres reported the employment of one or more physicians, with a median WTE of 0.95 (range 0.05-4.0). Twelve centres reported a median of 0.75 WTE of nurses (range 0.04-2.0).

**Figure 1:**
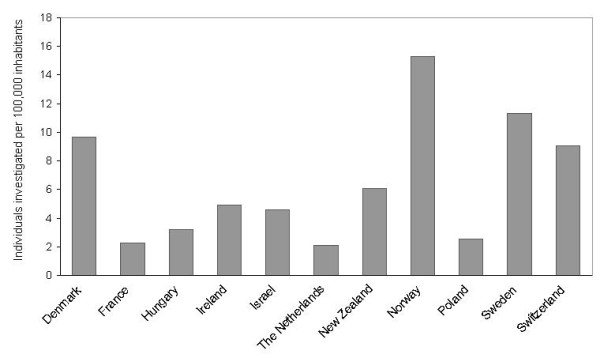
**Numbers of individuals investigated per 100,000 inhabitants in 2009.** Numbers of individuals investigated per 100,000 inhabitants in 2009 in countries where EPNET porphyria centres or associate members reported relevant data and national coverage of porphyria diagnostics.

In 2009, twelve centres in eleven countries reported that they provide national coverage of porphyria diagnostics in their respective countries (in Hungary by combination of two centres) and also reported the numbers of individuals they had investigated this year. The number of individuals investigated included both suspected new cases of porphyria as well as monitoring of known patients. The numbers of individuals investigated per 100,000 inhabitants in these countries ranged from less than three in France, Poland and the Netherlands to 15 in Norway (Figure 1).

**Figure 2:**
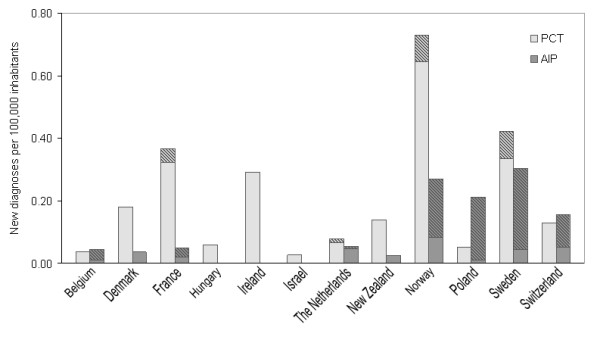
**New diagnoses of PCT and AIP per 100,000 inhabitants in 2009.** New diagnoses of porphyria cutanea tarda (PCT) and acute intermittent porphyria (AIP) per 100,000 inhabitants in 2009 in countries where EPNET porphyria centres or associate members reported national coverage of porphyria diagnostics, shaded areas representing presymptomatic cases.

The numbers of new diagnoses made per 100,000 inhabitants of the most common porphyrias, AIP and PCT, also varied greatly between centres with reported national coverage of porphyria diagnostics in 2009 (Figure 2). The highest rates of new PCT diagnoses (presymptomatic plus symptomatic) per 100,000 inhabitants were found in Norway (0.73), Sweden (0.42) and France (0.37). Sweden and Norway also had the highest numbers of new AIP diagnoses per 100,000 inhabitants (0.30 and 0.27, respectively), followed by Poland (0.21).

The proportion of presymptomatic PCT diagnoses relative to all new PCT diagnoses was low in all countries, whereas greater variation in symptomatic versus presymptomatic cases of new AIP diagnoses was evident. Some centres reported almost exclusively presymptomatic new cases of AIP, whereas others reported only new symptomatic cases.

**Figure 3:**
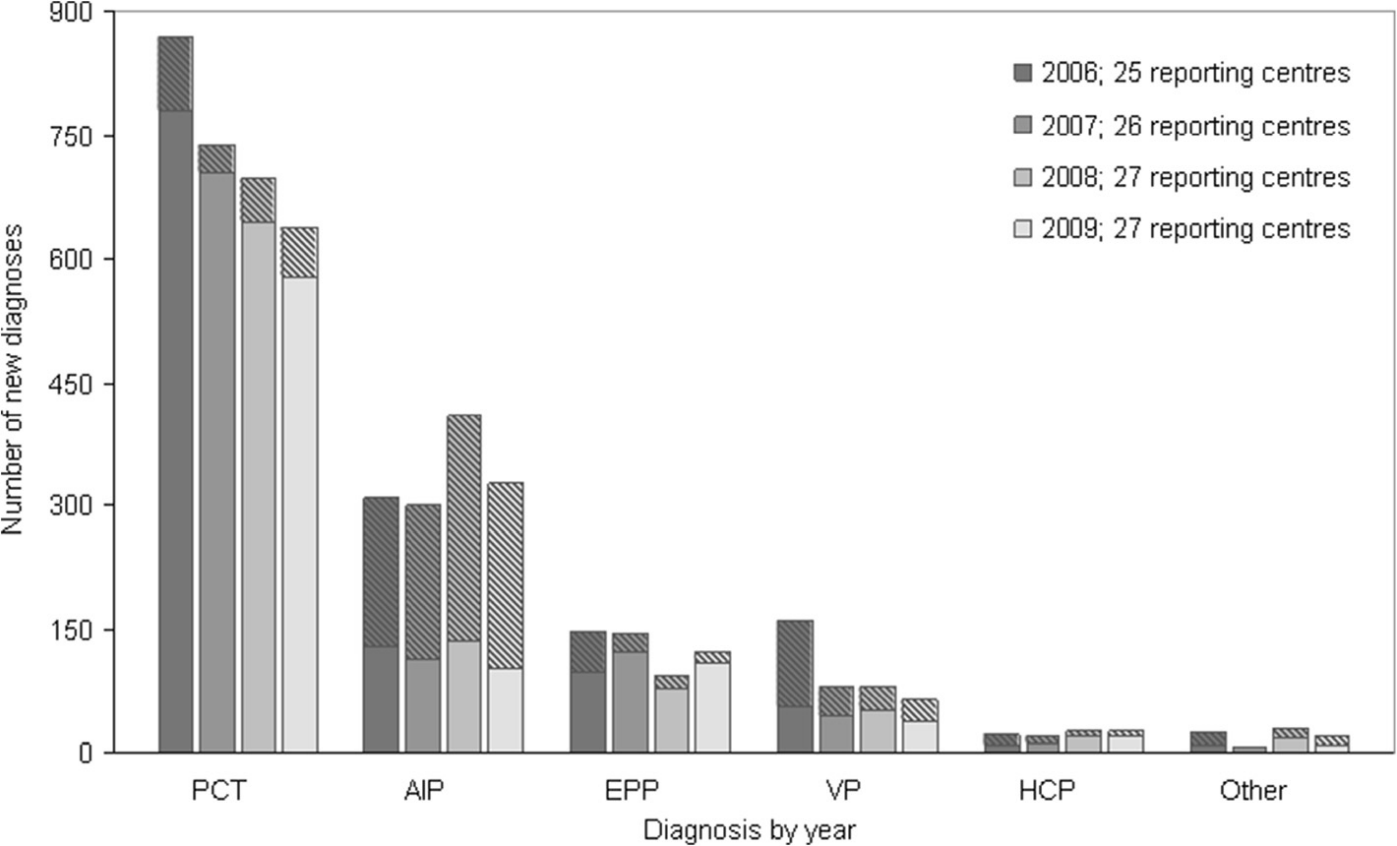
**Reported new porphyria diagnoses in Europe from 2006 to 2009.** Reported numbers of new porphyria diagnoses in Europe from 2006 through 2009, shaded areas representing presymptomatic cases.

With regard to new porphyria diagnoses reported in Europe from 2006 through 2009, PCT was the most frequently diagnosed porphyria overall (Figure 3), while AIP was the most frequently diagnosed acute porphyria. The proportion of new AIP diagnoses consisting of presymptomatic cases increased from 58% to 69% during the four year period. In 2006, 15 centres reported presymptomatic AIP cases, with nine centres reporting more presymptomatic than symptomatic cases. In 2009, 17 centres reported new presymptomatic AIP cases, of which 11 centres reported more presymptomatic than symptomatic cases. Ten centres reported new presymptomatic cases of PCT in 2006, compared to nine in 2009. The proportion of presymptomatic diagnoses of PCT remained close to 10% throughout the four years.

The proportion of EPNET centres and associate members’ laboratories participating in EQA schemes increased from 47% in 2006 to 85% in 2009 (Table 3). The proportion of centres reporting to have accredited or certified laboratory facilities increased from 47% in 2006 to 67% in 2009. In 2006, 24 out of 30 participating centres (80%) fulfilled the minimum criteria for being classified as a specialist porphyria centre. By 2009, 94% fulfilled the criteria.

**Table 3:** Number of porphyria centres fulfilling the minimum analytical and clinical criteria set by EPNET to be classified as specialist porphyria centres, reporting participation in external quality assurance schemes (EQAS) and having accredited and/or certified laboratory facilities

	**2006**	**2007**	**2008**	**2009**
	**(n = 30)**	**(n = 32)**	**(n = 32)**	**(n = 33)**
Fulfilling minimum criteria	24^a^	27^a^	32^a^	31^a^
Participation in EQAS for metabolites and/or enzymes^b^	14	20	26	28
Accredited and/or certified laboratory^c^	14	17	20	22

^a^Two centres were considered specialist porphyria centres in conjunction with another centre.

^b^Part of the minimum criteria from 2009 onwards. Reported EQA schemes:

Royal College of Pathologists of Australasia Quality Assurance Programs (RCPA-QAP), European Porphyria Network External Quality Assessment Scheme (EPNET EQAS), Welsh External Quality Assurance Scheme (WEQAS), Society for Promotion of Quality Assurance in Medical Laboratories (INSTAND e.V.), Quality Control Centre Switzerland (CSCQ), Dutch Foundation for Quality Assessment in Clinical Laboratories (SKLM).

^c^Reported standards: ISO 9001, ISO 14001, ISO 17025, ISO15189, CPA (UK), CAP/CLIA, ISO 9000, ISO 17089.

## Discussion

The porphyrias are rare diseases that can be complicated to diagnose, and in most European countries the full spectrum of porphyria diagnostics is offered by national or specialized porphyria laboratories or diagnostic centres. Since 2001, many such porphyria centres have been cooperating under the EPI/EPNET, with non-European centres participating as associate members, striving to adhere to agreed quality criteria. This study shows that in 2009 there were large variations between porphyria centres with regard to the numbers and type of laboratory analyses performed, workload and dedicated personnel available. However, from 2006 through 2009 there was evidence of improvements in the analytical repertoire offered by participating centres. More importantly, increasing numbers of participating centres fulfilled the minimum criteria for being classified as an EPNET specialist porphyria centre; by 2009 94% fulfilled them.

As a part of EPNET’s WP6, the EPNET EQA scheme was established in 2008. Twice a year, biological specimens from a porphyria patient are distributed along with a case history to participating laboratories. The scheme covers diagnostic strategies, analytical laboratory performances, the clinical interpretation and reporting of results. Evaluation of the programme revealed large variations with regard to analytical and diagnostic performances [7]. However, regular participation rapidly led to improvements in diagnostic strategies and more uniformly use of standardized units. The present study confirms that from 2006 through 2009, there was evidence of improvements in analytical laboratory services offered. The differentiation between the faecal coproporphyrin isomers I and III is of particular importance when diagnosing or excluding the rare HCP [6], and more centres could perform this analysis over time, and did so increasingly. Similarly, an increasing number of centres was able to distinguish between metal free and zinc-chelated protoporphyrin. This is of importance when diagnosing EPP [8], particularly in patients with a low erythrocyte protoporphyrin concentration and when differentiating between XLDPP caused by a mutation in ALA synthase 2 gene [9] and EPP caused by mutations in the ferrochelatase gene. Also, more centres offered plasma fluorescence scanning during the study period. This analysis is necessary to discriminate between both cutaneous and acute porphyrias, and of particular importance when diagnosing VP.

Having observed that more centres were able to perform essential laboratory analyses over time, we sought to investigate if centres that had introduced new analyses had in fact diagnosed and reported more cases of the relevant porphyrias. We were, however, not able to confirm any such trends (data not shown). HCP, EPP and VP are rare porphyrias of which most centres identify only a few new cases each year, some years none. Making the correct diagnosis in a symptomatic porphyria patient is a complex process, where offering the correct laboratory analyses is an essential, but not sufficient prerequisite. The clinician first has to suspect porphyria and send adequate biological samples to a diagnostic centre. After having performed and interpreted correctly the necessary laboratory analyses, the diagnostic centres can confirm or exclude the diagnosis. With such small numbers of new diagnoses reported each year, it is not surprising that an observed trend of improvement in laboratory analyses offered alone is not enough to show an improved ability to diagnose certain porphyrias. Still, we believe this to be an important step in the right direction for better care for porphyria patients.

In addition to the observed improvements in laboratory analyses offered by EPNET centres and associate members, improvement was evident from the increasing proportion of centres fulfilling the minimum criteria for being classified as a specialist porphyria centre, laboratories participating in EQA schemes and reporting to have accredited or certified facilities. The increased awareness of the porphyrias caused by the efforts of EPI/EPNET, the yearly activity feedback reports that may serve as a reminders of best practise and turn attention to areas where each centre might focus their efforts to improve their services, and the establishment of the EPNET EQA scheme in 2007 [7] may be, at least in part, responsible for the observed improvements.

A considerable variation in numbers of individuals investigated per 100,000 inhabitants was observed between the 11 countries where participating centres reported that they provide national coverage of porphyria diagnostics, ranging from less than three to 15. The numbers of individuals investigated included both suspected new cases of porphyria as well as monitoring of known patients. The observed variation could therefore partly reflect the clinicians’ varying awareness of the porphyrias and willingness to send biological samples for testing. Also, routines for follow-up and monitoring of known patients differ between countries. For instance, where recommendations are made to analyse yearly control samples from patients in remission, as well as monitoring more frequently when symptoms occur, as in Norway, a relatively larger number of individuals investigated per 100,000 inhabitants are to be expected. Where recommendations for follow-up are not the same, not agreed upon or not widely practised, other numbers would be expected.

Large differences in the numbers of new diagnoses of AIP and PCT per 100,000 inhabitants were also evident. A certain degree of diversity in the occurrence of the porphyrias between countries is, however, to be expected. Most mutations of porphyria are private, but several founder mutations have been described, contributing to founder effects and higher occurrence of disease in geographic regions [10-13]. Also, the variation probably reflects differences in resources used to investigate healthy at-risk relatives with DNA testing. In some countries, reported new diagnoses of acute porphyrias consisted almost exclusively of presymptomatic cases, while other centres did not offer presymptomatic testing at all. In addition, in some countries or areas, intense case finding projects have already been performed, leaving fewer new cases to be diagnosed, which could also contribute to the observed differences.

In Europe, the proportion of new cases of PCT that were presymptomatic as opposed to symptomatic remained close to 10% throughout the study period, while the proportion of presymptomatic cases of AIP increased from 57% to 69%. The benefits of presymptomatic testing of healthy at-risk relatives in the acute porphyrias are obvious; knowledge about carrier status of a DNA mutation allows a person to avoid factors known to provoke acute attacks. Preventing acute attacks can improve quality of life, reduce the risk of potentially life threatening situations and reduce long term morbidity [1]. The proportion of centres performing DNA-analyses did not increase during the study-period, suggesting perhaps a potential for identifying more at–risk relatives. For the cutaneous porphyria PCT, however, the benefits of presymptomatic DNA testing are more questionable. Although the cutaneous symptoms of PCT can be troublesome, uncomfortable and often long-lasting, they are not life-threatening and can in most cases be treated by repeated phlebotomies or low dose chloroquine treatment [1]. In addition, a clinically indistinguishable form of non-inheritable PCT exists where the same environmental factors are necessary to cause disease (iron excess, hepatitis C infection, alcohol, oestrogens) [12]. Thus, there is a risk of developing PCT independent of DNA mutation carrier status.

It is important to note that the reported numbers of new diagnoses in this study are hampered by uncertainties and should not be interpreted as figures of incidence. Presumably, many cases of the cutaneous porphyrias such as PCT, and to some extent EPP, are diagnosed and treated by dermatologist with little involvement from specialist porphyria centres, and thus never reported to EPNET. The reported national coverage of porphyria diagnostics is therefore thought to be more accurate for the acute porphyrias (ALA dehydratase deficiency, AIP, HCP and PV) than for the cutaneous porphyrias. Even so, a tendency towards overestimating the numbers of new acute porphyria diagnoses made may be present, as it cannot be ruled out that the same patient may occasionally be reported from more than one centre. On the other hand, centres that do not offer presymptomatic DNA testing probably report relatively lower numbers of new diagnoses. It is also likely that in certain regions and countries porphyria services could be substandard or lacking so that symptomatic patients are not diagnosed. True incidence rates of clinically overt inherited porphyrias in European countries have recently been investigated under the EPNET’s WP7 [14].

The minimum criteria for classification as a specialist porphyria centre set by EPNET are continuously evaluated and improved when considered necessary. For instance, participation in a relevant EQA program was introduced as a minimum criterion in 2009, and being able to differentiate between metal free and zinc-chelated protoporphyrin was added in 2010. At present, it is a concern that some centres perform only a limited amount of some or all laboratory analyses offered each year, which may compromise both the quality and the interpretation of the analyses. Therefore, some requirements regarding the numbers of yearly analyses performed may also, in the future, be included as part of the minimum criteria for classification as a specialist porphyria centre.

## Conclusions

This study confirms that in 2009, the distribution of porphyria services was uneven among EPNET centres and associate members. However, improvements in the quality of diagnostic services were observed during 2006 through 2009. The improvements may at least in part be attributable to the cooperation between the participating centres, the continuous evolvement of and agreement upon best practice in the field, and the feedback reports provided to the centres by EPNET, serving as reminders of best practise. The challenges for the future are first and foremost to further improve the services of existing EPNET porphyria centres, to recruit and help improve services of porphyria centres not already participating in the EPNET, a priority of EPNET at present, and to encourage the establishment of porphyria centres in countries or regions lacking in such services today. In addition, it is crucial to continue raising awareness of these rare diseases to clinicians outside the field. Improvements in porphyria laboratory and diagnostic services are of little use to patients unless clinicians use them.

## Abbreviations

AIP: Acute intermittent porphyria; ALA: δ-aminolevulinic acid; PCT: Porphyria cutanea tarda; EPP: Erythropoietic protoporphyria; XLDPP: X-linked dominant protoporphyria; CEP: Congenital erythropoietic porphyria; HCP: Hereditary coproporphyria; VP: Variegate porphyria; EPI: European Porphyria Initiative; EPNET: European Porphyria Network; EQA: External quality assessment; PBG: Porphobilinogen; WP: (EPNET) work package; WTE: Whole time equivalent.

## Competing interests

The authors declare that they have no competing interests.

## Authors’ contributions

MT participated in design of the study, interpretation of data and drafted the manuscript. AA, JHV and ES participated in design of the study, interpretation of data and revised the manuscript critically. JCD, JM and JTO participated in acquisition of data and revised the manuscript critically. SS participated in conception and design, acquisition and interpretation of data and revised the manuscript critically. All authors have given final approval of the published version.
